# Pulmonary Hemosiderosis in Children with Bronchopulmonary Dysplasia

**DOI:** 10.1155/2014/876195

**Published:** 2014-09-17

**Authors:** David Kurahara, Marina Morie, Maya Yamane, Sarah Lam, Wallace Matthews, Keolamau Yee, Kara Yamamoto

**Affiliations:** Department of Pediatrics, John A. Burns School of Medicine, University of Hawaii, Honolulu, HI, USA

## Abstract

We describe a possible association between pulmonary hemosiderosis (PH) and a history of bronchopulmonary dysplasia (BPD). Both patients were born at 28-week gestation and presented with PH at ages 22 months and 6 years, respectively. Both initially presented with cough and tachypnea, and bronchoalveolar lavage showed evidence of hemosiderin-laden macrophages. Initial hemoglobin levels were < 4 g/dL and chest radiographs showed diffuse infiltrates that cleared dramatically within days after initiation of intravenous corticosteroids. In the first case, frank pulmonary blood was observed upon initial intubation, prompting the need for high frequency ventilation, immediate corticosteroids, and antibiotics. The mechanical ventilation wean was made possible by the addition of mycophenolate mofetil (MMF) and hydroxychloroquine. Slow tapering off of medications was accomplished over 6 years. These cases represent a possible correlation between prematurity-associated BPD and PH. We present a review of the literature regarding this possible association. In addition, MMF proved to be life-saving in one of the PH cases, as it has been in pulmonary hemorrhage related to systemic lupus erythematosus. Further studies are warranted to investigate the possible association between PH and prematurity-related BPD, as well as the use of MMF in the treatment of PH.

## 1. Introduction

Pulmonary hemosiderosis (PH) can present with a catastrophic lung hemorrhage in previously healthy young children. It has rarely been described to occur in children with previous lung disease like bronchopulmonary dysplasia (BPD). In idiopathic pulmonary hemosiderosis (IPH) no underlying cause can be found and it can present with iron-deficiency anemia, recurrent hemoptysis, and diffuse parenchymal infiltrates on chest radiograph (CXR) [1]. Iron-deficiency anemia results due to hemosiderin iron deposition in the alveoli [2]. Recurrent episodes of alveolar hemorrhage may occur and the clinical course of IPH is variable. The finding of hemosiderin-laden macrophages (HLM) by bronchoalveolar lavage (BAL) is helpful to confirm the diagnosis [3]. IPH most commonly affects children, although adult cases have been reported [1]. IPH has been treated with corticosteroids and/or immunosuppressive drugs with variable success [1, 2]. Here we investigate a possible association between PH and history of prematurity-associated bronchopulmonary dysplasia (BPD), as well as the utility of mycophenolate mofetil (MMF) as a treatment option for PH.

## 2. Case History

### 2.1. Case 1

The patient was a 22-month-old Filipino female born at 28-week gestation at a weight of 1,073 g. Neonatal course was complicated by BPD, grade I intraventricular hemorrhage (IVH), retinopathy of prematurity (ROP), and necrotizing enterocolitis requiring a sigmoid resection. She was discharged home at 3 months of life at a weight of 3000 g and required one year of oxygen therapy without further complications.

At 22 months of age, she presented to the Emergency Department with hemoglobin of 3.5 g/dL and fluffy infiltrates on CXR. Upon immediate transfer to the pediatric intensive care unit, intubation was attempted, but gross red blood was noted to be coming out of the endotracheal tube (ET). Intubation was reattempted and ET placement was confirmed visually, but gross red blood continued to flow from the ET. CXR showed diffuse pulmonary infiltrates and bronchoscopy revealed abundant HLM which was helpful in the diagnosis of PH. She was put on high frequency ventilation and started on intravenous (IV) methylprednisolone (2 mg/kg/d) and broad spectrum antibiotics. At this point she was critically ill and it was felt that a lung biopsy was not possible. She improved dramatically and was successfully weaned to a conventional ventilator in 2 days, then continuous positive airway pressure (CPAP) 7 days later. She was not able to be tapered off of CPAP until the addition of MMF upon pediatric rheumatology consult. PH work-up including antinuclear (ANA), antineutrophil cytoplasmic (ANCA), antiglomerular basement, cardiolipin, cow's milk preciptins, gliadin, and reticulin antibodies was all negative. Urinalysis and complement studies were also normal. After successful CPAP wean, she was discharged home after 1 month of hospitalization.

Over the next 2 years, she had recurrent episodes of respiratory distress requiring hospitalization. Each episode showed diffuse pulmonary infiltrates on CXR and rapid improvement upon IV corticosteroid administration. When hydroxychloroquine (HC) was added to her medication regimen, she stabilized significantly and has not required further hospitalizations.

Her medications were slowly tapered off over the next 6 years: corticosteroids first, then MMF, and finally the HC. Follow-up CXRs, hemoglobin, pulmonary function tests, and growth velocity have all normalized.

### 2.2. Case 2

The patient was a 6-year-old Filipino female born at 28-week gestation, with neonatal course complicated by BPD requiring oxygen therapy for 15 months, congenital tracheal stenosis and tracheal ring s/p reconstructive surgery, grade II IVH, developmental delay, ROP, and neurosensory hearing loss s/p cochlear implant. After an initial prolonged hospitalization in the neonatal ICU, she did well until she presented with intermittent cough and rapid breathing for 2 weeks. She had tactile fever and tachycardia but no history of hemoptysis, hematemesis, or hematochezia.

In the emergency room she was tired and pale with mild hypoxia and conjunctival pallor. Complete blood count showed likely iron-deficiency anemia (hemoglobin = 3.3 g/dL, hematocrit = 11.9, MCV = 77.2, iron = 21, TIBC = 531, and reticulocyte count = 14.8). CXR showed bilateral pulmonary infiltrates. In addition, CRP = 0.1 and ESR = 24.

With suspicion of PH, she was given a packed red blood cell transfusion, diuretics, and IV corticosteroids (2 mg/kg/d). BAL showed abundant HLM which was useful in the diagnosis of PH. Due to prior tracheal ring surgery and the history of BPD it was felt that lung biopsy would have put her at high risk for complications so this was not performed. Further work-up revealed elevated ANA of 320 (nl < 40), positive SSA/Ro titer of 99.6 (nl < 20), and positive antimyeloperoxidase ANCA (anti-MPO) of 11.5 (nl < 9). The rest of her ANA studies, anti-GBM, cow's milk precipitins, gliadin, and reticulin antibodies were negative. Her Hb stabilized with IV corticosteroids, no further bleeding occurred, and she was discharged home after one week. Parents were counseled on the use of other immunosuppressant drugs in PH but decided against these unless she had another episode. Her corticosteroids were slowly tapered off over 4 years with no further episodes of bleeding and normal CXRs and pulmonary function tests. Her anti-MPO antibody disappeared after the initial positive finding. ANA and SSA/Ro antibodies remain positive and continue to be followed by pediatric rheumatology, but she has not fulfilled criteria for SLE.

## 3. Discussion

Very few cases in the literature describe a history of prematurity in children who develop PH. However, there may be an unrecognized association between prematurity-related BPD and later development of PH. One case in the German literature describes IPH in a 3-year-old child with history of ductus ligation at 32 weeks and birth weight of 1405 g [4]. Another article describes a male, born at 32-week gestation, diagnosed with IPH at 2 years of age [2]. There is no mention of BPD during the neonatal course, but BPD was less likely to develop given a 32-week gestational infant.

Could earlier lung damage have predisposed these children to develop PH later in life? BPD is defined as the need for supplemental oxygen for ≥ 28 days of life [5]. Both of our cases fulfill the criteria for BPD. BPD is characterized by diffuse airway damage and alternating areas of overinflation with atelectasis, fibrosis, smooth muscle hypertrophy, and prominent vascular hypertensive lesions with airway wall thickening [5]. As children with BPD get older, they have higher rates of wheezing, pneumonia, long-term medication use, and respiratory symptoms and higher hospitalization rates than controls without BPD [6–8]. Therefore, prior history of BPD and its associated lung damage may predispose to increased risk of recurrent pulmonary hemorrhage and lower respiratory tract infections. Similarly, a 9-year-old male developed postinfectious bronchiolitis obliterans with PH, confirmed by lung biopsy and CT scan, following a viral infection at 7 months of age with years of persistent respiratory symptoms [9]. Further studies are needed to delineate a link between prematurity-related BPD and IPH. A history for any child suspected to have IPH should include a detailed birth history.

The treatment for IPH has been well described to include corticosteroids and immunosuppressive agents [2] as this illness includes both inflammatory and immune components. Hydroxychloroquine has been used successfully [10] and in our first case proved to be helpful in reducing bleeding episodes, which prevented further hospitalizations. In a series of 26 children with IPH by Kabra et al., symptoms did not recur in 17 individuals initially started on both prednisolone and HC [10]. A retrospective study looking at prognosis of IPH showed improved outcome with long-term immunosuppression via corticosteroids, HC, and/or azathioprine [11].

MMF is used increasingly in severe SLE and other autoimmune diseases [12]. It has been lifesaving in retractable pulmonary hemorrhage refractory to corticosteroids, cyclophosphamide, and even plasmapheresis, which usually carries a very high mortality rate of 70–95% [13–15]. MMF is a potent immunosuppressive agent that inhibits proliferation of T and B lymphocytes and suppresses antibody formation by B lymphocytes, without hepatotoxicity, nephrotoxicity, or mutagenicity [16]. It is an immunosuppressive agent used in transplantation, with evidence of superior protection against acute transplant rejection compared to azathioprine-containing regimens [12]. This medication proved effective in our first case. It allowed for successful wean from the ventilator after a very severe pulmonary hemorrhage. It was felt that monthly pulse cyclophosphamide would not be effective rapidly enough to avoid a fatality, and the side effects of daily cyclophosphamide would surpass those of daily MMF. No published reports of MMF used in IPH were found, but we feel that it was instrumental in our patient's improvement. Further studies are needed, but MMF may provide added immunosuppression during life-threatening pulmonary hemorrhage in IPH and other autoimmune disorders.

Our cases illustrate the often-difficult decision of obtaining a lung biopsy on an actively hemorrhaging lung. Both cases were started on corticosteroids prior to pulmonary and rheumatology involvement, so it was felt that biopsy risk would outweigh the benefit, especially on two children with a previous history of BPD and possible lung scarring. Earlier literature describes the importance of getting a lung biopsy to rule out other vasculitic illnesses [2, 3]. However, more recent literature supports making the diagnosis using BAL [1, 10, 17].  Calculating the percent of HLM may improve the sensitivity to 100% and the specificity to 96% when an index of 35% or more is used [17]. Biopsy may be helpful in some cases of protracted pulmonary disease as in the case with bronchiolitis obliterans described by Pinto et al. [9]. The logistics and risk of lung biopsy in a child with an active pulmonary hemorrhage should be considered carefully.

Previous chronic lung disease may be a risk factor for the development of PH. We report two cases of PH in children with history of prematurity-related BPD. Since they had previous lung damage related to prematurity they were classified as PH as the idiopathic description is a diagnosis made from exclusion of other possible coexistent lung diseases. Further studies are needed to investigate this association, and detailed birth histories should be elicited for all cases of suspected IPH. In one case, the use of MMF and hydroxychloroquine was helpful in improving the disease outcome. MMF may be a useful agent in PH management as seen in SLE-related pulmonary hemorrhage.
